# Comparison of Tubal Sterilization Procedures Performed by Keyless Abdominal Rope-Lifting Surgery and Conventional CO_2_ Laparoscopy: A Case Controlled Clinical Study

**DOI:** 10.1155/2013/963615

**Published:** 2013-12-24

**Authors:** Kahraman Ülker, Ürfettin Hüseyinoğlu

**Affiliations:** ^1^Department of Obstetrics and Gynecology, School of Medicine, Kafkas University, 36000 Kars, Turkey; ^2^Department of Anesthesia and Reanimation, School of Medicine, Kafkas University, 36000 Kars, Turkey

## Abstract

*Objective*. To evaluate the safety and efficacy of Keyless Abdominal Rope-Lifting Surgery (KARS), for tubal sterilization procedures in comparison with the conventional CO_2_ laparoscopy. *Material and Methods*. During a one-year period, 71 women underwent tubal ligation surgery. Conventional laparoscopy (*N* = 38) and KARS (*N* = 33) were used for tubal sterilization. In KARS, an abdominal access pathway through a single intra-abdominal incision was used to place transabdominal sutures that elevated the abdominal wall, and the operations were performed through the intraumbilical entry without the use of trocars. In CO_2_ laparoscopy, following the creation of the CO_2_ pneumoperitoneum a 10 mm trocar and two 5 mm trocars were introduced into the abdominal cavity. Tubal sterilizations were performed following the creation of the abdominal access pathways in both groups. The groups were compared with each other. *Results*. All operations could be performed by KARS without conversion to CO_2_ laparoscopy or laparotomy. The mean operative time of the two groups was not significantly different (*P* > 0.05). Intra- and postoperative findings including complications, bleeding, and hospital stay time did not differ between groups (*P* > 0.05). *Conclusion*. KARS for tubal sterilization seems safe and effective in terms of cosmesis, postoperative pain, and early hospital discharge.

## 1. Introduction

Surgical sterilization as a safe and reliable permanent contraceptive method is preferred by more than 190 million couples worldwide and 36% of fertile women (about 700,000 annually) in the USA choose this method among all contraceptives [1, 2]. Ligation of the Fallopian tubes may be performed at the time of delivery, shortly after delivery or at another time (interval sterilization). Between 1994 and 1996 half of the sterilizations were performed as interval procedures in the USA [3].

Laparoscopic tubal sterilization may be performed at anytime other than the immediate postpartum period. Laparoscopic approach requires a 5 or 10 mm umbilical camera port and a secondary port to introduce various instruments. In some cases, in order to handle the tubes, bowel, or possible adhesions an additional port is also required. In most of the cases, in addition to the umbilical port, two 5 mm ports are needed.

The laparoscopic tubal ligation is relatively contraindicated in patients with severe cardiopulmonary dysfunction. In addition, the risks of laparoscopic procedures are related to abdominal cavity access techniques (50% of the major complications like gastrointestinal and major blood vessel injuries) [4], creation of pneumo-peritoneum, use of intra-abdominal energy, and increased anesthesia risks [5–9]. Thus, the researchers have tried and published new laparoscopic techniques in order to find the most efficacious manner of minimizing the side effects and maximizing the advantages of laparoscopic surgery [10–15].

Recently, a novel, gasless, single-incision abdominal access technique (keyless abdominal rope-lifting surgery, KARS) was defined for the management of benign ovarian cysts [16–18]. In this study we aimed to compare the tubal sterilization procedures performed by KARS and conventional CO_2_ laparoscopy.

## 2. Materials and Methods

### 2.1. Patients and Data Evaluation

This retrospective study was approved by the Ethics Committee of Kafkas University School of Medicine. The study included operations performed between May 2010 and May 2011 within the obstetrics and gynecology department of Kafkas University School of Medicine. However, the data evaluation continued till the second half of 2013. All included women were recontacted and informed consents were provided. In order to analyze the contraception failure rates all participants were reevaluated after completing the second postoperative year.

The included women were allocated into two groups as the study group and the control group. The study group consisted of the women operated by using the new single-incision, gasless laparoscopy technique (keyless abdominal rope-lifting surgery, KARS) and the control group consisted of the women operated by using the conventional CO_2_ laparoscopy during the same period.

The study included the women requested for surgical sterilization without a remarkable health problem.

The demographic and physical characteristics of the participating women included the age, gravidity, and parity of the patients as well as the number of the abortions, ectopic pregnancies, and offspring. The height, weight, and the body mass index of the patients were also obtained.

Following the use of KARS technique in 2010 in order to collect specific and detailed data the total operative times and abdominal cavity access times had been recorded in patients' charts. The abdominal cavity access time contained the time needed for the construction of the pathway into the abdominal cavity and the rope-lifting process during KARS and the time needed for the creation of the CO_2_ pneumo-peritoneum and the insertion of the three intra-abdominal trocars during conventional laparoscopy.

All the participating women were re-contacted in 2013 and invited to have an interview and examination. During the examination the surgical site was examined and a questionnaire including the contraceptive failure, menstrual changes, and additional health problems was filed. In case where the women were not able to come to the examination, the interviews were completed by telephone conversations.

### 2.2. Patient Preparation

One day prior to the operation, oral intake was prohibited at 11:00 p.m.; however, the participants were allowed to drink liquids until the last four hours before the scheduled surgery. On the surgery day a mixture of 19 g sodium dihydrogen phosphate and 7 g disodium hydrogen phosphate in 135 mL solution was used rectally at 6 am before the surgery.

The patients were prepared in the supine decubitus position in the operating theatre between 8:30 and 10.30 a.m.

### 2.3. Surgical Principles

KARS procedures have been performed within the obstetrics and gynecology department of Kafkas University School of Medicine since 2010. We have used the technique in the surgical management of various benign gynecologic conditions. The KARS procedure was used to create an access pathway into the abdominal cavity and to elevate the abdominal wall in order to provide an operative space.

**Figure 1:**
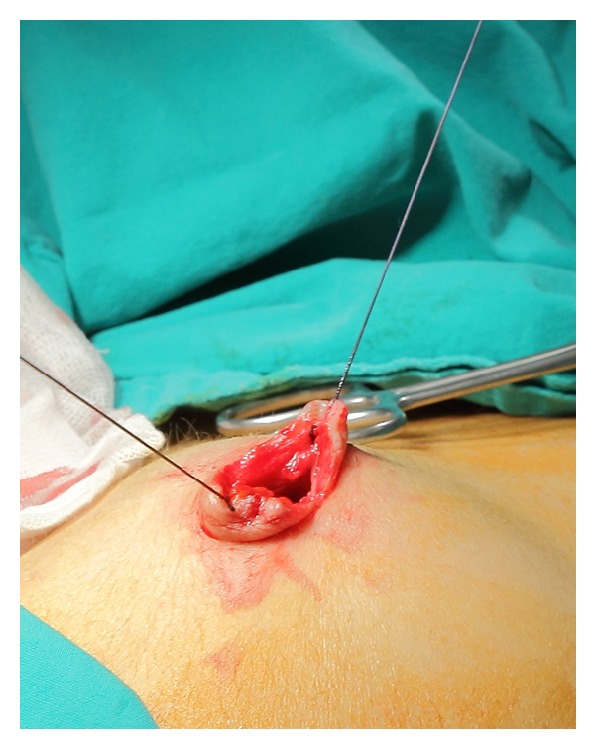
For lifting the entry side, the facial layer of the 1–1.5 cm midumbilical *transverse incision *was sutured with a number 0 delayed-absorbable suture from the lower and upper border of the incision at 6 and 12 o'clock positions, respectively.

The details of the KARS technique can be found somewhere else [16–18]. In summary, following the mechanical and chemical cleaning of the abdominal wall and the umbilical region, a 1.5–2 cm *transverse incision* including the facial layer was performed at the centre of the umbilicus. For lifting the entry side, the facial layer underlying the incision was sutured with a number 0 delayed-absorbable suture from the lower and upper border of the incision at 6 and 12 o'clock positions, respectively (Figure 1). A universal ether screen at the level of the line between the superior anterior iliac crests was maintained 10–12 cm above the abdominal surface and covered with sterile drapes.

**Figure 2:**
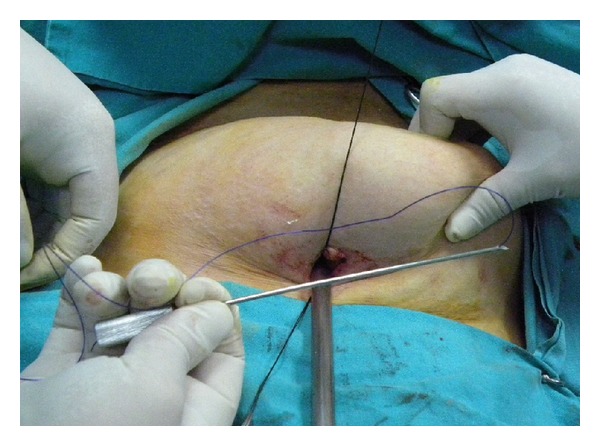
The preparation of the Veress cannula. The needle of the Veress cannula was removed, and one tip of a no. 0 nylon suture was inserted approximately 8–10 cm into the Veress cannula.

**Figure 3:**
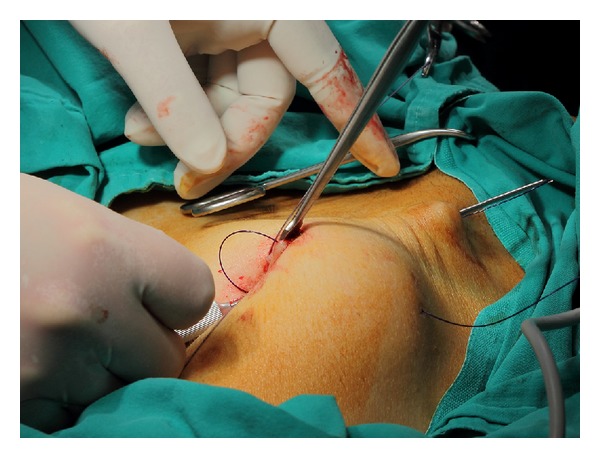
Transabdominal passage of the Veress cannula loaded with a nylon suture.

**Figure 4:**
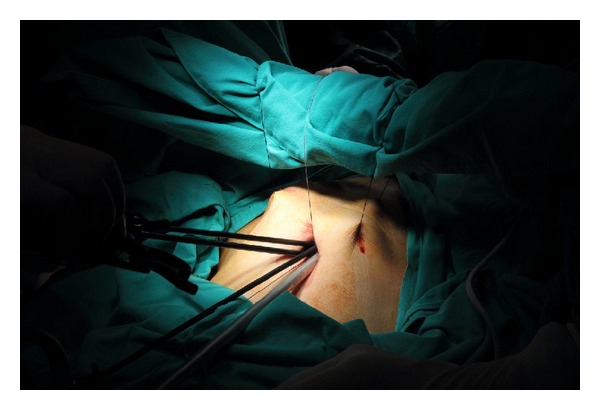
The abdominal wall was elevated by an assistant, and the surgeon tied the sutures over the preprepared sterile ether screen. Multiple instruments without their trocars can be used in the same single incision.

The needle of the Veress cannula was removed, and one tip of a no. 1 nylon suture was inserted approximately 8–10 cm into the Veress cannula (Figure 2). The loaded cannula was introduced into the elevated entry site. The tip of the cannula perforated the parietal peritoneum one to two cm lateral to the entry and it was slid over the peritoneum. The loaded cannula was oriented laterally right or left 6-7 cm to avoid injury to the epigastric vessels. By using the sharp tip of the Veress cannula, the abdominal wall was pierced from the inside toward the outside, and the suture was unloaded from the cannula outside the abdominal wall. After removing the unloaded cannula back from the entry by loading of the second suture tip into the cannula, the same procedure was performed 5-6 cm below the first tip's entry side. The same procedure was repeated at the contra lateral side of the abdominal wall (Figure 3). Once the lifting sutures were ready, the abdominal wall was elevated by an assistant, and the surgeon tied the sutures over the preprepared ether screen or retractor (Figure 4).

The abdominal access process for conventional CO_2_ laparoscopy included 5 mm infraumbilical skin incision, insertion of the Veress needle into the peritoneal cavity, testing the needle location, insufflations of CO_2_ until reaching an intra-abdominal pressure of 12–14 mmHg, enlargement of the skin incision to 11 mm, insertion of a 10 mm trocar, application of the telescope and under direct vision creation of two additional 5 mm ports at 5 cm lateral and 5 cm below the first entry site.

### 2.4. The Tubal Sterilization Procedure

Following the construction of the abdominal access pathways and the operative fields in each group, the Fallopian tubes were handled by a laparoscopic grasper and then a 0.5 to 1.5 cm portion of the tube was coagulated with a laparoscopic bipolar cautery. The coagulated portion was cut and detached by using a laparoscopic scissor to complete the partial salpingectomy. During the sterilization procedures the uterus was elevated by the help of a vaginally introduced manipulator.

### 2.5. Statistical Analysis

The data were analyzed by using SPSS for windows version 16.oo. Means, medians, and standard deviations were used for descriptive statistics. The characteristics of the two operative groups were compared with each other. In order to study the learning curves the first 15 cases were compared with the sequent cases of their groups. The characteristics with normal and nonnormal distributions were compared by using Student *t*- and Mann Whitney tests, respectively. Spearman's correlation test was used to evaluate the relationship between the study parameters. A *P*  value of <0.05 was considered significant.

## 3. Results

The study included 71 tubal sterilization procedures, 33 KARSs, and 38 conventional CO_2_ laparoscopies, in a one-year period. Three of the women (one in KARS and two in conventional laparoscopy groups) did not come to visit in 2013 and completed their interview with telephone conversation.

There were no intraoperative complications in any of the 71 cases other than minor bleedings. All of the operations could be performed without conversion to conventional laparoscopy or laparotomy in KARS group and without conversion to laparotomy or minilaparotomy in conventional laparoscopy group. Simple oral analgesics following the postoperative immediate 50 mg meperidine IM were adequate for postoperative pain relief in both groups.

The data (Table 1) including the demographics, physical characteristics, and preoperative findings of the participating women did not differ between groups (*P* > 0.05).

The intra-operative and post-operative findings (Table 2) were similar in both groups (*P* > 0.05); however, the abdominal cavity access time in KARS group was significantly longer than that in the conventional laparoscopy group (*P* < 0.05). The mean operative and abdominal cavity access times were significantly decreased (Table 3) following the first 15 cases in both groups (*P* < 0.05).

Correlation analysis showed that the demographic variables like age, gravidity, parity, and offspring numbers correlated strongly with each other (*P* < 0.05) but not with the intra- or post-operative findings.

In KARS group there were strong positive correlations among the abdominal access and rope-lifting process time, the whole operation time, the body mass index of the women, and the hemoglobulin and hematocrit drop rates (*P* < 0.05). However, the post-operative hospital stay time did not correlate with any of the abovementioned parameter (*P* > 0.05).

In conventional CO_2_ laparoscopy group there were strong positive correlations among the abdominal access and the whole operation times, the body mass index of the women, and the hemoglobulin and hematocrit drop rates (*P* < 0.05). However, the post-operative hospital stay time did not correlate with any of the abovementioned parameter (*P* > 0.05).

During the visit 2013 no contraceptive failure was recorded. The intraumbilical scars were hardly identifiable and there were not any incisional hernias. Two women in KARS group and three women in CO_2_ laparoscopy group described a milddecrease in their menstrual blood flow volume.

## 4. Discussion

### 4.1. Principal Findings

In this study, we were able to perform tubal sterilization on 33 women by using a single-incision, gasless laparoscopic technique, KARS. The abdominal access pathway was maintained through the intraumbilical 1.5–2 cm incision and all operations were successful without the need for conversion to conventional laparoscopy or laparotomy. The means of operation time and abdominal cavity access time decreased after performing the first 15 cases. At the end of the second year none of the participants experienced a failure of tubal sterilization performed by KARS or conventional CO_2_ laparoscopy.

### 4.2. Strengths of the Study and KARS

This is the first study on tubal sterilization describing a gasless single-incision laparoscopic technique in which the intra-abdominal vision is maintained following the elevation of the abdominal wall by using surgical ropes. All the operations were performed by the same surgical team and under the same operative theatre conditions. Although the study included a limited number of participants, the included groups had similar demographic, physical, and medical characteristics.

One third of the complications of CO_2_ laparoscopy occur during pneumo-peritoneum creation or trocar instillation by the blind introduction of the Veress needle or trocars into the abdominal cavity [9]. Because KARS is an open laparoscopic technique, the chance of visceral injury occurring is minimal.

KARS is a gasless laparoscopic technique which protects the patient from the pneumo-peritoneum associated side effects like hypercapnia, acidosis, gas embolism, pneumothorax, subcutaneous emphysema, deep venous thrombosis, instability of the hemodynamics, decrease in renal functions, and peritoneal oxidative stress [5–7, 10, 11, 19–21]. Gasless surgery is also more optimal for regional anesthesia. However, longer operative time and patient position limit the use of regional (spinal and epidural) anesthesia in KARS [16].

In CO_2_ laparoscopy, trocars with valves are needed to maintain the created pneumo-peritoneum during surgery. Laparoscopic hand instruments are used through trocars to prevent gas leakage and maintain intra-abdominal high pressure [22]. However, in KARS the abdominal wall is elevated with surgical ropes, and neither the intra-abdominal gas nor the trocars for maintaining vision of the operative field are required. Trocars generally need a 1 mm larger diameter (20% for a 5 mm diameter and 10% for a 10 mm diameter) than the instruments employed, and the extra abdominal part of the trocars, containing valves and locks, are even larger. In contrast, KARS' trocar-less entry, by sparing more space, allows for multiple instrument entry through the same single access route. In addition, the hand instruments do not need to fit with anything other than the incision. This characteristic also allows the use of conventional surgical instruments used in conventional laparotomy. Moreover KARS allows demanded amounts of irrigation and aspiration without decreasing the intra-abdominal space blurring the vision.

The fixed working envelope around each port often necessitates multiple ports to accommodate changes in instrument position for improved visibility and efficiency. However, additional ports contribute to post-operative pain, diminish cosmesis, and carry a risk of bleeding, hernia formation, or organ damage. In addition, the special ports used in single incision surgeries have limited access holes. One of the access holes is for the telescope, and in general there are only two holes for hand instruments [15, 23, 24]. KARS is a single-incision surgery and has all of the advantages of the single-incision surgery. In this manner, KARS also has the advantage of permitting the use of multiple instruments at the same time. (During some operations we used 3 hand instruments and the telescope at the same time.)

In conventional laparoscopy the ports are rather too small in diameter to handle them with conventional surgical instruments and thus some surgeons do not close the facial layer of the abdominal wall if 10 mm trocars are used. Almost all surgeons do not close the facial layer if 5 mm trocars are used which may lead to hernia formation. However in KARS the facial layer is prepared for closure at the initial stage of the construction of the abdominal access pathway. The fascia is elevated and stitched easily as in usual open surgery.

### 4.3. Limitations of the Study and KARS

Although our retrospective study included groups with similar demographic characteristics (*P* > 0.05, Table 1), it lacks the power of a prospective randomized study.

This retrospective case controlled study was performed in a single center by the same surgical team. Although it is useful for creating a homogeneous perioperative condition to compare the findings of both surgical techniques, the comparison of the surgical teams with different levels of surgical skills is lacking.

At the beginning of 2010 laparoscopic surgery was not a routine application for the surgical management of gynecologic disorders in our department. Initially we built a laparoscopic surgery team and began to perform laparoscopic surgery. Although the team members had some experience on laparoscopic surgery, the harmony among individual team members was lacking. Thus, the team's surgical skills and harmony were not at an advanced level for KARS and/or conventional laparoscopy.

The limited number of cases was not adequate to study appropriately the learning curve of KARS (Table 3). Although, following the first 15 cases, the means of operation time and abdominal cavity access time decreased, the same decreases were also observed in conventional CO_2_ laparoscopy group. This finding might represent a better orientation of the surgical team to both operation techniques in time. In order to better study the learning curve of KARS the study should be repeated by a better-organized and more experienced surgical team.

Because the women with previous abdominal operations and dense intra-abdominal adhesions were excluded, our study cannot evaluate the feasibility of KARS in women with dense intra-abdominal adhesions.

Our study reflects the results of tubal sterilization at the end of the second year. However the rate of the success of the tubal sterilization in a longer time period is not known. In addition, the number of included women (*N* = 71) is not sufficient for conclusions that can be applied to the whole population. The reader should note that the study mainly deals with the operative techniques.

KARS has the same common disadvantages of any single-incision laparoscopic surgery like sword fighting of the instruments and the telescope, obstruction of the operative field by a hand instrument passing in front of the telescope, and the difficulty of the manipulation of the instruments introduced parallel. In addition, the trocar-less entry causes more frequent contamination from incision edges, and the telescope needs to be cleaned more frequently [16].

In KARS, the partial elevation of the upper abdominal viscera results in a smaller space which may cause difficulties to hold most of the bowel out of the operative field adequately. Our previous study demonstrated that increasing the upper abdominal space with the stitches placed through the overlying skin of the supraumbilical region removes the bowels to the upper abdominal cavity adequately [16]. However, we did not need to use the additional sutures during tubal sterilizations. The vaginally placed uterine manipulator provided the uterine elevation, thus the adequate visualization of the tubes.

KARS is a modified technique that employs the features of laparoscopy and laparotomy which necessitates the familiarity of the surgeons with both techniques. However, the abdominal access technique has similarities with the laparotomy and is conducted under direct vision. The elevation procedure is simple and any surgeon familiar to laparotomy can perform it easily and swiftly after a few procedures.

### 4.4. Comparison with the Previous Studies and Techniques

Beginning from 1993 gasless (isobaric) laparoscopic surgery has been defined and used for a wide variety of gynecological surgical procedures [25, 26] such as myomectomy [27–29], hysterectomy [18, 30], ovarian cyst resection [16–18, 31], colposuspension [32], and radical hysterectomy [33]. In all operations special surgical instruments, such as a special device with an abdominal retractor, a subcutaneous lifting device, or an airlift balloon retractor, have been used to elevate the abdominal wall. However, in KARS only the usual conventional surgical materials and instruments were used for the lifting process [16–18].

Laparoendoscopic single-site (LESS) surgery is used to describe various surgical techniques that aim to perform laparoscopic surgery through a single incision [23]. LESS has gained world-wide popularity since 2005 and various operations including gynecological cancer staging, salpingo-oophorectomy, ovarian cystectomy, laparoscopy assisted vaginal hysterectomy, and laparoscopic total hysterectomy have all been performed by using LESS [33–38]. LESS procedures used two (or more) conventional ports or a single multichannel device (which enables the passage of instruments and optics) placed in a single incision. Although some multichannel devices had ports for three instruments and an optic [39], most of the time the conventional trocars and multichannel devices allow for only a limited numbers of instruments to be used. In contrast, with its trocar-less access property KARS provided more space for instruments and the optic within a similar-sized incision. In addition, it spares the additional cost of the special access ports. Moreover, the conventional surgical instruments (with stronger jaws) fitting with the incision could also be used if needed [16–18].

The mean operative time of laparoscopic tubal sterilizations varied between 20 and 25 minutes [40, 41]. It might depend on the subjective conditions of the operative theatre and the subjective qualification of the surgical team. In our study, the mean operation time for conventional laparoscopic tubal sterilization and KARS was not significantly different (*P* > 0.05) as 22.42 ± 6.55 and 27.76 ± 12.62 minutes, respectively (Table 2). However the mean abdominal cavity access times of 11.71 ± 2.95 for conventional laparoscopy and 15.45 ± 6.71 for KARS were significantly different (*P* < 0.05). The discordance of the findings may result from the easier closure of the abdominal access pathway in KARS. The preprepared facial sutures helped to close the umbilical incision of KARS. In contrast, the facial suturing and closure was harder in conventional laparoscopy.

During laparoscopic sterilization the tubes may be occluded by various methods including electrosurgical methods using unipolar or bipolar electrocoagulation or mechanical methods such as the Hulka-Clemens spring clip, the Filshie hinged clip, or the Falope or Yoon silastic ring/band [42]. US Collaborative Review of Sterilization Study (CREST) [43] found that the efficacy of tubal sterilization varied by the patient's age, race, and ethnicity. In addition, the lowest postprocedural pregnancy rates were achieved following unipolar coagulation and postpartum partial salpingectomy. Bipolar tubal coagulation was also found highly effective where the tubal coagulation was adequate [44]. In our study, we performed a partial salpingectomy by using laparoscopic bipolar cautery and scissor, and a 5 to 10 mm portion of the tube was removed. At the end of the second year none of the women got pregnant.

Depending on the findings of our study KARS procedure seems feasible for tubal sterilizations. However, the reader should note that the limited number of the participants and time of the follow-up period of our study necessitate further prospective studies consisting of larger serious with longer follow-up periods.

## 5. Conclusion

KARS is a gasless, single-incision laparoscopy technique and seems safe and effective in terms of cosmesis, postoperative pain, and early hospital discharge for tubal sterilization. It allows the use of laparoscopic and conventional instruments and does not depend on trocars and special multichannel devices which may significantly decrease the cost of single-incision laparoscopic technique.

## Figures and Tables

**Table 1:** The demographics, physical characteristics, and the preoperative findings of the participating women in both groups. The data was presented with mean ± standard deviation or median values.

Characteristic	KARS (*n* = 33)	CO_2_ laparoscopy (*n* = 38)	*P* value
Maternal age	37.06 ± 3.73	35.66 ± 3.05	0.091*
Gravidity	6	5	0.314*
Parity	4	4	0.639*
Miscarriage	0	0	0.592**
Induced abortion	1	1	0.553*
Ectopic pregnancy	0	0	0.283**
Offspring number	4	4	0.600*
Mean height (cm)	161.82 ± 4.47	162.42 ± 4.83	0.587*
Mean weight (kg)	69.42 ± 10.21	67.89 ± 10.73	0.541*
Mean body mass index (kg/m^2^)	26.63 ± 4.55	25.89 ± 4.86	0.511*
Mean initial hemoglobulin (gr/dL)	12.59 ± 1.46	12.57 ± 1.37	0.952*
Mean initial hematocrit (%)	37.72 ± 3.39	37.61 ± 3.32	0.890*

*Student's *t*-test (used for normal distribution), **Mann Whitney *U* test (used for nonnormal distribution).

KARS: Keyless Abdominal Rope-lifting Surgery.

CO_2_ laparoscopy: Conventional multiport laparoscopy performed following the creation of pneumoperitoneum.

**Table 2:** The intraoperative and postoperative findings of the study according to the operative techniques. The data was presented with mean ± standard deviation values.

Parameter	KARS (*n* = 33)	CO_2_ laparoscopy (*n* = 38)	*P* value
Mean operation duration (min)	27.76 ± 12.62	22.42 ± 6.55	0.114**
Mean abdominal cavity access time (min)	15.45 ± 6.71	11.71 ± 2.95	*0.016∗∗ *
Mean final hemoglobulin (gr/dL)	11.64 ± 1.29	11.67 ± 1.21	0.909*
Mean final hematocrit (%)	34.70 ± 3.30	34.79 ± 3.15	0.912**
Mean hemoglobulin drop (gr/dL)	0.95 ± 0.54	0.90 ± 0.53	0.672*
Mean hematocrit drop (%)	3.02 ± 1.67	2.81 ± 1.67	0.606*
Mean postoperative hospital stay (days)	0.39 ± 0.50	0.52 ± 0.56	0.293*

*Student's *t*-test (used for normal distribution), **Mann Whitney *U* test (used for nonnormal distribution), KARS: Keyless Abdominal Rope-lifting Surgery.

CO_2_ laparoscopy: conventional multiport laparoscopy performed following the creation of pneumoperitoneum, italic *P* values indicated the significant values.

**Table 3:** The comparison of the first 15 operations with the later operations in both surgical techniques. The data was presented with mean ± standard deviation values.

		First 15 cases	Following cases	*P* value*
KARS (*n* = 33)	Mean operation time (min)	35.80 ± 12.18	21.05 ± 8.55	0.001
	Mean abdominal cavity access time (min)	19.60 ± 6.67	12.00 ± 4.50	0.001
CO_2_ Laparoscopy (*n* = 38)	Mean operation time (min)	27.27 ± 5.32	19.26 ± 5.26	<0.001
	Mean abdominal cavity access time (min)	13.33 ± 1.84	10.65 ± 3.08	0.005

*Student's *t*-test (normal distribution), KARS: Keyless Abdominal Rope-lifting Surgery.

CO_2_ laparoscopy: conventional multiport laparoscopy performed following the creation of pneumoperitoneum.
